# Multiplexed Passive Optical Fiber Sensor Networks for Water Level Monitoring: A Review

**DOI:** 10.3390/s20236813

**Published:** 2020-11-28

**Authors:** Hoon-Keun Lee, Jaeyul Choo, Joonyoung Kim

**Affiliations:** 1 Department of Safety Research, Korea Institute of Nuclear Safety, 62 Gwahak-ro, Yuseong-gu, Daejeon 34142, Korea; hklee@kins.re.kr; 2 Department of Electronics Engineering, Andong National University, 1375 Gyengdong-ro, Andong-si, Gyeongsangbuk-do 36729, Korea; jychoo@anu.ac.kr; 3 Department of Smart Information Communication Engineering, Sangmyung University, 31 Sangmyungdae-gil, Dongnam-gu, Cheonan-si 31066, Korea

**Keywords:** water management, water level monitoring, passive optical fiber sensor network, quasi-distributed, multiplexing

## Abstract

Water management is a critical mission required to protect the water resources that is essential in diverse industrial applications. Amongst a variety of parameters such as level (or depth), temperature, conductivity, turbidity, and pH, the water level is the most fundamental one that needs to be monitored on a real-time basis for securing the water management system. This paper presents an overview of water level monitoring technologies based on optical fiber sensor (OFS) networks. Firstly, we introduce and compare the passive distributed and quasi-distributed (discrete) sensor networks with the recent achievements summarized. The performance (i.e., sensing range and resolution) of the OFS networks can be enhanced through diverse multiplexing techniques based on wavelength, time, coherence, space, etc. Especially, the dense wavelength division multiplexing (DWDM)-based sensor network provides remote sensing (where its reach can be extended to >40 km) with high scalability in terms of the channel number that determines the spatial resolution. We review the operation principle and characteristics of the DWDM-based OFS network with full theoretical and experimental analysis being provided. Furthermore, the key system functions and considerations (such as the link protection from physical damages, self-referencing, management of sensing units, and so on) are discussed that could be a guideline on the design process of the passive OFS network.

## 1. Introduction

A water quality management technology is crucial in a variety of fields (from the industrial to the public sectors) [1,2]. In general, it requires data collection on a regular basis through in-field sampling and the following lab-based analysis [1]. However, it is costly and time-consuming to gather the data and draw a meaningful information through the analysis. In addition, the transportation of the samples from the field to the remote laboratory could lead to undesirable physical/chemical perturbations. More importantly, the sampling and collection of data occur not quite often (for example, monthly or seasonally), making the agile reaction difficult, especially in an emergency. Therefore, a real-time water monitoring system is essential in various fields to remotely analyze the various parameters as to the water quality.

The water quality data typically include various physical/chemical parameters, such as level, temperature, conductivity, turbidity, pH, and so on. Among those, the water level is the fundamental information that most of the water systems require to monitor [3]. For example, in a nuclear power plant, the water level must be monitored and controlled for the spent fuels to cool down and, also, to shield the radiation fields. In spite of the recent reinforcement of the water-related regulations, the water level monitoring systems still remain costly, complex, and inconvenient to install/implement. Thus, in recent years, a remote-sensing network based on optical fiber sensors (OFS) has been attracting the growing interests of many different fields, such as energy, security, civil infrastructure, and so on [4,5,6]. This is due to the various advantages of the OFS, such as a passive sensing capability without the necessity of electrical power feeds. The OFS also has broadband and low-loss characteristics that are attributed to the properties of optical and photonic devices. It subsequently allows for a remote sensing (e.g., >10 km) and resistances to electromagnetic interference and radiations. Moreover, various multiplexing techniques can be exploited to enhance the performance as compared to conventional technologies, such as capacitive sensors [7] and resistive sensors [8]. To be more specific, in response to the fourth industrial revolution, the sensing system is also being required to increase its data capacity that the multiplexed optical fiber sensor network can readily realize [1].

This paper reviews the water (or liquid) level monitoring systems realized by the OFS networks. Firstly, distributed and quasi-distributed (discrete) passive OFS networks are discussed with the recent works introduced in Section 2. Then, we introduce and compare several state-of-the-art water level monitoring systems based on diverse multiplexing techniques (i.e., wavelength, time, and coherence) with discrete sensing units (SU). The recent development progress with more detailed characteristics of the multiplexed OFS networks are introduced in Section 3. Following that, in Section 4, we specifically review the dense wavelength division multiplexing (DWDM)-based technology that we recently proposed to support tens of channels within the Telecom C-band. Finally, we discuss critical design factors of a passive OFS network, such as protection, self-referencing, SU management, etc. in Section 5. Note that the main focus of this paper is on the multiplexed OFS networks using arrays or sets of on-off sensors rather than the systems that utilize distributed sensors or a single sensor, e.g., based on a direct pressure measurement [9,10].

## 2. Passive Quasi-Distributed Optical Fiber Sensor Network for Water Level Monitoring

**Figure 1:**
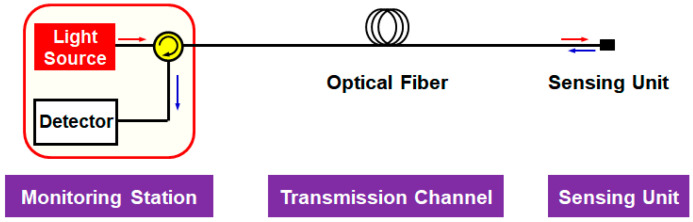
Basic architecture of a passive optical fiber sensor network (point-to-point).

The basic architecture of the passive optical fiber sensor network (point-to-point) is shown in Figure 1 [11].

This structure is generally divided into three function blocks: (i) a monitoring station, which includes an interrogation system, (ii) the transmission channel that comprises an optical fiber, and (iii) a SU for measuring environmental changes. An additional function block can be added between the optical fiber and SU, being a remote node (that includes, e.g., arrayed waveguide grating (AWG) or power splitter) for multiplexing signals from multiple SUs. The interrogation system comprises an optical source such as a distributed feedback-laser diode (DFB-LD) or a broadband light source (BLS) for seeding the light into the network, a detector such as photo-diode (PD) or an optical spectrum analyzer (OSA) for interrogating the reflected signal, and an optical circulator (OC) for separating the transmission and detection parts. As a transmission media, a single-mode fiber (SMF) is commonly employed where its length would be tens of kilometers. The SU is installed at the measurement point, embarking the environmental information on the light wave with, e.g., fiber Bragg grating (FBG) [12] or the Fabry-Perot interferometer (FPI) [13] employed as one of the discrete sensors. This architecture enables parameter (i.e., the water level) monitoring at a remote place without the power supply to the measurement point.

### 2.1. Optical Fiber Sensor Network Architectures

**Figure 2:**
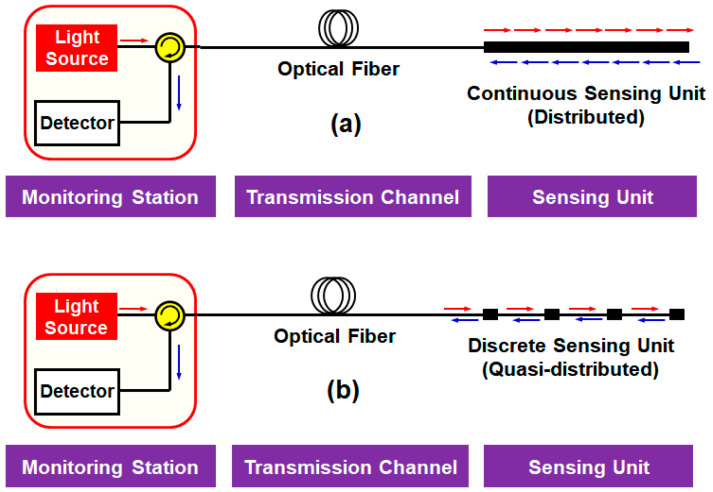
Classification of optical fiber sensor network architectures (point-to-multipoint) according to the spatial distribution of the measurement points: (**a**) distributed and (**b**) quasi-distributed.

There are various criteria to classify the type of sensor networks: the network topology (bus or star); transducing approach (intrinsic or extrinsic); and modulation mechanism (intensity, frequency, phase, and so on) [14]. On the other hand, the OFS networks can be categorized into two different groups according to the spatial distributions of the measurement points, as shown in Figure 2: (a) distributed and (b) quasi-distributed (i.e., discrete).

The distributed sensors perform continuous sensing within a specific area/range at relatively high spatial resolutions that are realized by a Rayleigh-, Brillouin-, or Raman scattering-based optical time-domain reflectometer (OTDR) [6,11,15], which is the basic mechanism of fault localization in fiber optic communications. In the sensor applications, the OTDR techniques enable to detect the spatial variation of the measurand, thus allowing it to be profiled at a given spatial resolution within the length of the utilized optical fiber. These methods have a trade-off between the spatial resolution and the measurement range [16]. For example, the Raman-based OTDR provides either the high spatial resolution (1 cm) for the short sensing range (<1 km) [17] or the long sensing range (<37 km) at relatively low spatial resolution (17 m) [18]. Moreover, complex signal processing is needed, increasing the cost and processing time [15,19].

On the other hand, the quasi-distributed sensor networks perform the sensing at multiple discrete points at which the SUs are installed (Figure 2b). The use of multiple discrete SUs, which were implemented with optical filters such as FBG [12] and FPI [13], enhanced the resolution, sensing range, and reliability. To further improve the system practicability, researchers embedded the multiple SUs into a low-cost plastic optical fiber (POF) [20,21,22,23,24,25,26,27,28,29], as summarized in Table 1. The basic principle lies in the fact that the bending loss of the multimode POF varies according to the medium surrounding the fiber [20,21]. Some researches laterally polished the bends of the POF to improve the sensitivity, each bend acting as the SU [22,23]. More recently, the twisted flat POF was proposed, achieving the highest sensitivity of 0.52 dB/mm [24]. Other POF-based sensors exploit the variation of the fiber’s transmittance (i.e., the output power) according to the water level. To achieve this, the POF with periodic micro-holes [25] and multi-POF segments [26] were proposed. Then, the transmittance increased when the fiber was immersed in the water, since the refractive index of the water was closer to that of the fiber core as compared to the air. For the same purpose, the POF with V-shaped grooves were utilized, by which the output power linearly increased with the water level [27,28,29].

### 2.2. Multiplexing Techniques for the Quasi-Distributed Optical Fiber Sensor Network

In the quasi-distributed OFS network, the multiplexing allows a single interrogation system (i.e., a single optical source and a single detector) to support multiple SUs, enhancing the system performance, as well as the practicability (in terms of install and operation costs/efforts) [14,30]. As summarized in Table 2, various multiplexing techniques have been proposed for the OFS network with discrete SUs [31,32,33,34,35,36,37,38,39,40,41,42,43].

**Figure 3:**
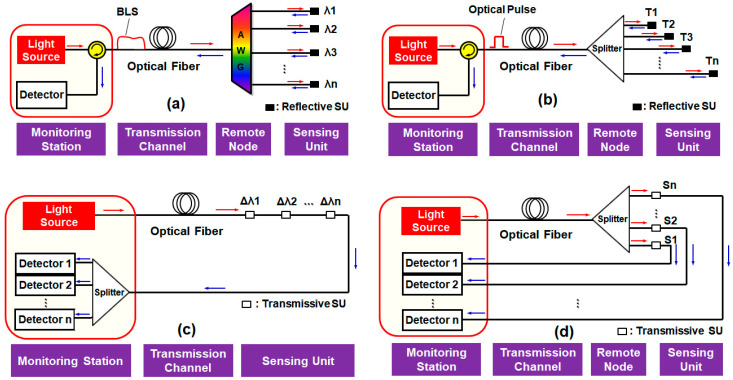
Basic architectures for various multiplexing techniques: (**a**) wavelength division multiplexing (WDM), (**b**) time division multiplexing (TDM), (**c**) coherence division multiplexing (CDM), and (**d**) space division multiplexing (SDM). SU: sensing unit and AWG: arrayed waveguide grating. λ*n*, *Tn*, Δλ*n* and Sn represent the channel numbers of the allocated wavelength, time, coherence length and space for multiplexing/demultiplexing, respectively.

The multiplexing techniques can be categorized into (a) wavelength division multiplexing (WDM), (b) time division multiplexing (TDM), (c) coherence division multiplexing (CDM), and (d) space division multiplexing (SDM), as shown in Figure 3.

Firstly, the WDM is one of most popular multiplexing techniques in telecommunications, especially in the optical access network [44]. In WDM-aided OFS networks, each SU utilizes a dedicated wavelength (i.e., channel) that is determined by a multiplexer/demultiplexer (i.e., AWG). Then, the optical signals from the multiple SUs are multiplexed, as shown in Figure 3a, which provides good scalability and network transparency. The implementation of the WDM system relies on the wavelength-independent (i.e., color-free) optical sources that are realized by arrayed DFB-LD [45], multimode Fabry-Perot laser [46], amplified spontaneous emission (ASE) light [47], multi-wavelength fiber lasers [14], and wavelength tunable laser sources [48,49]. The WDM can be further divided into two groups according to the channel spacing: (i) coarse WDM (CWDM) and (ii) dense WDM (DWDM). The CWDM system typically utilizes the FBG as a SU due to its inherent advantages, such as robustness and high linearity [30]. However, it requires enough guard band to avoid crosstalk that restricts the maximum number of channels. Recently, on the other hand, a DWDM-based all-optical water level monitoring system has been reported to support more than 32 channels with 100-GHz channel spacing [42,43,50], as will be discussed in Section 4.

The TDM is one of the most conventional multiplexing techniques, though still in frequent use, that features high simplicity and efficiency. As Figure 3b describes, each SU is allocated to a particular time slot for sending information [30], generating a periodic (i.e., burst mode) pulse train towards the monitoring station. TDM comprises a single optical pulsed source, detector (configuring OTDR system), and a power splitter for the interrogation of multiple SUs. However, the insertion loss of a 1 × *N* optical coupler (i.e., splitter) increases with the number of multiplexed channels, and therefore, there exists a restriction on the number of SUs. Moreover, it requires fine control of the transmission delay through a complicated link design process [51] or using optical delay coils [52].

Along with TDM, the CDM is another earlier investigated multiplexing technique. This is based on a coherent light source and several interferometric SUs. These SUs are interrogated by utilizing different degrees of mutual coherence with respect to the reference carrier, i.e., according to the different optical path difference (OPD), as shown in Figure 3c [53]. Moreover, the sensor network based on CDM needs careful designing so the OPD of each interferometric SU is longer than the coherence length of the light source. Here, additional interferometric SUs are employed as detectors at the monitoring station, matching the path mismatch of each SU and providing coherent interference. However, the number of SUs is limited by excess phase-to-intensity conversion noise, leading to a poor signal-to-noise ratio (SNR) [53].

In the SDM, each SU sends the signal to the monitoring station via a dedicated fiber optic cable, so-called fiber multiplexing [30]. It requires multiple laser sources with a single interrogator, as shown in Vázquez et al. [32]. As shown in Figure 3d, however, a single optical source can be employed to reduce the system cost and complexity. Then, the optical output power is divided by the optical splitter to multiple SUs that restrict the number of channels. Furthermore, the dedicated fiber optic connection for the multiple SUs would increase the total cost.

Additionally, a combination of two multiplexing techniques could be considered to overcome the limitations each technique has. This approach is generally referred to as a hybrid multiplexing technique [14,30]. A hybrid WDM/TDM scheme is the most frequently used, in which each wavelength (i.e., each WDM channel) forms the TDM network [51,54]. For example, the FBG array offers a large-scale sensing capacity with, e.g., >1000 sensing points. However, its spatial resolution is relatively large (>2 m) due to the pulse width of the light source [51], and the transmission length is quite limited by Rayleigh scattering [11] and/or multiple-reflection crosstalk [54], being unsuitable for the application of remote water level monitoring.

The various multiplexing techniques have specific advantages and limitations. Thus, one can consider specific aspects (e.g., the modulation mechanism, sensing range, the number of SUs, interrogation method, and transmission length) for specific applications. Nevertheless, the DWDM system provides various applications with diverse merits, as we will discuss in Section 4.

### 2.3. Remote-Sensing Capability of the Quasi-Distributed Optical Fiber Sensor Network

The remote-sensing system based on the OFS network has been attracting growing attention in recent years, since its high practicability manifested itself in many different applications. To be specific, the passive remote-sensing architecture allows to monitor important parameters from a monitoring station located more than tens of kilometers away from the field (in which SUs are installed) without the electrical power feeds. It consequently allows for immediate damage detection and quick responses. However, the conventional long-reach OFS networks based on a loop-back architecture suffer from signal degradation induced by the Rayleigh back-scattering (RBS) of the seeded light source [11].

To overcome this problem, several approaches were proposed, one of those based on a heterodyne detection in conjunction with radio frequency (RF) up-conversion [55,56]. It can enhance the SNR, though costly and complex to implement. Another solution employs a high-speed wavelength sweeping with using a tunable laser source [57]. The RBS is considerably mitigated through turning off the optical source for a while after every single sweep. The other solution utilized two separate transmission paths [50]: one is for seeding the light source, and the other is for receiving the optical signals from the SUs. The detail of the last solution is presented in Section 4.2.

## 3. Recent Development in Multiplexed Optical Fiber Sensor Networks

In this section, we further explain the details of the passive OFS networks based on three representative multiplexing techniques (TDM, SDM, and CWDM), comparing their pros and cons.

### 3.1. TDM-Based Water Level Monitoring System

**Figure 4:**
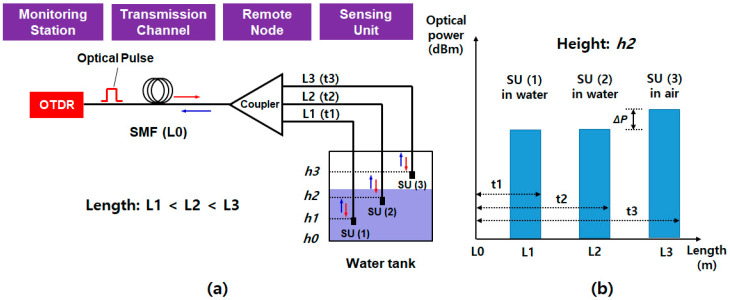
(**a**) Schematic diagram of the TDM-based water level monitoring system and (**b**) conceptual interrogation result at the water level of *h*2. Adapted from Yoo et al. [36]. OTDR: optical time-domain reflectometer, SMF: single-mode fiber, SU: sensing unit, *L*0: fiber length of SMF, *L*1–*L*3 (*t*1–*t*3): fiber lengths (travelling times) according to the channels, and *h*0–*h*3: height of the water level where the SU are installed.

The TDM-based water level monitoring system is illustrated in Figure 4a [36]. It consists of the OTDR unit, SMF (the transmission channel), the optical coupler in the remote node, and multiple SUs in the water tank. The TDM technique employs a single optical source and a single detector (i.e., forming the OTDR link) to interrogate multiple SUs, thus significantly simplifying the demodulation electronics [30]. The optical pulse from the OTDR is sent to the SMF with the length of L0 (e.g., 50 cm in Yoo et al. [36]), its power equally distributed by the 1 × *N* optical coupler (splitter). The output of the coupler is connected to the SUs with fibers of difference lengths (e.g., L1 of 3 m, L2 of 5 m, and L3 of 10 m, respectively). The SUs are installed in the water tank, as seen in Figure 4a, where each SU is specially fabricated based on the optical patch cord with the NaCl solution (sodium chloride 99.5% plus distilled water), which is contained in the stainless-steel case [36]. This case is attached at the end of the patch cord with a rubber sealing ring to avoid the leakage of the solution. The OTDR operates based on the Fresnel reflections of the optical pulses at the interface between the optical fiber end and the NaCl solution. This aqueous solution has high thermal conductivity, changing its own refractive index (i.e., changing the reflected optical power by the power difference (ΔP), as shown in Figure 4b) according to the water level. Figure 4b presents the conceptual interrogation result at the water level of *h*2. In this case, SU (1) and SU (2) are submerged in the water, while SU (3) is not. Then, the reflected lights are displayed on the OTDR monitor as a function of the traveling time (or length) according to the designed fiber lengths that are connected to the SUs.

The TDM scheme can be easily implemented with off-the-shelf optical components, except the specially fabricated SUs [36]. Moreover, it has multiparameter sensing capabilities, i.e., measuring both the temperature and the water level at the same time, thanks to the special probes filled with the NaCl solution. However, the variation range of the NaCl solution index is still not enough; thus, the power difference (ΔP) is less than 1 dB for the water temperature range of 25–60 °C. It could lead to the incorrect reading of the water level. In addition, the insertion loss of the optical coupler increases with the number of SUs, not only restricting the spatial resolution but, also, further degrading the system performance. Moreover, as mentioned in Section 2.2, it requires fine control of the transmission delay through a complicated link design process [51] or using optical delay coils [52].

### 3.2. SDM-Based Water Level Monitoring System

**Figure 5:**
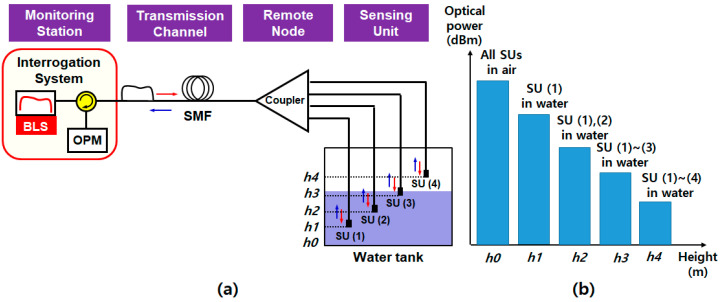
(**a**) Schematic diagram of the SDM-based water level monitoring system (**b**) Conceptual interrogation result according to water level. Adapted from Noor et al. [41]. BLS: broadband light source, OPM: optical power meter, SMF: single mode fiber, SU: sensing unit, and *h*0–*h*4: height of the water level where the SU are installed.

As shown in Figure 5a, the SDM system has a similar architecture to that of TDM, except that the interrogation system in the monitoring station uses the OPM instead of the OTDR unit [41]. To be specific, an amplified spontaneous emission (ASE) light output of the C-band BLS is divided by the 1 × *N* optical coupler at the remote node, where each channel’s output (of the optical coupler) is back-reflected at the end facet of the SU. Each SU is the fiber-optic cable with the flat-cleaved facet. Figure 5b presents the conceptual interrogation result of the various water levels: from *h*0 to *h*4. When the water level is *h*0 (i.e., nearly empty), the sum of the reflected power becomes the maximum, as the SUs in the air result in the larger reflections. However, the sum of the reflected optical power decreases as the water level rises. Thus, there is a linear relation between the measured optical power and the level of water, which could be used to estimate the actual water level [41].

The SDM system features the simple architecture that could be implemented with off-the-shelf optical components. Moreover, the use of an OPM makes the system highly cost-effective as compared to using the optical spectrum analyzer. However, this scheme needs reference power information for the correct measurement (the water level is determined by comparing the measured optical power to the reference power level). Moreover, Rayleigh back-scattering (RBS) at the SMF restricts the remote-sensing distance. In addition to this, similar to the TDM, there is a limitation to improve the spatial resolution due to the huge insertion loss of the optical coupler, especially for the large number of SUs.

### 3.3. CWDM-Based Water Level Monitoring System

**Figure 6:**
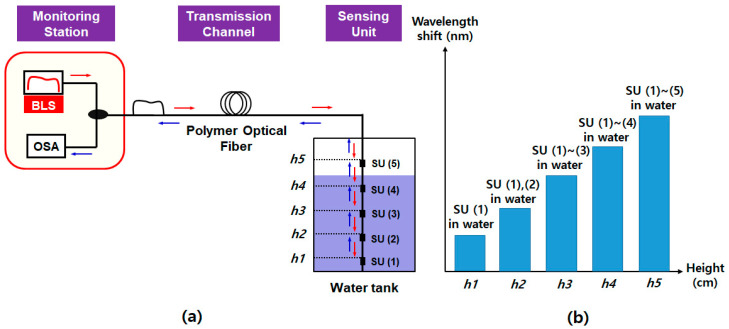
(**a**) Schematic diagram of the coarse wavelength division multiplexing (CWDM)-based water level monitoring system. (**b**) Conceptual interrogation results according to the water level. Adapted from Marques et al. [37]. BLS: broadband light source, OSA: optical spectrum analyzer, SU: sensing unit, and *h*1–*h*5: height of the water level where the SU are installed.

The water level monitoring system based on CWDM is illustrated in Figure 6a. This scheme is made up of an interrogation system (BLS plus OSA) in the monitoring station, polymer optical fiber as the transmission channel, and FBGs (SUs) in the water tank [37]. The output of the BLS (e.g., a super-luminescent diode at 830 nm with a 40-nm spectral width) is transmitted to the SUs that are micro-structured polymer optical FBG (mPOFBG). In Marques et al. [37], a single 75-cm polymer optical fiber was used, where five multiplexed mPOFBGs were inscribed with a spatial separation of 15 cm. This polymer fiber can provide a relatively low loss of 7 dB/m in an 850-nm band. The water level is determined by measuring the wavelength shift of the SUs (i.e., the FBG-based pressure sensors) at the bottom of the water tank. The wavelength shifts of the SUs are in linear relation to the depth. To facilitate this principle, each mPOFBG is combined with one diaphragm, and the FBGs are positioned at the center of each diaphragm. These diaphragms provide internal pressure by the water to the mPOFBGs for water level monitoring. Figure 6b presents the conceptual interrogation result of the SU (1) for the various water levels: from *h*1 to *h*5. When the water level is below the SU (1), i.e., below *h*1, no wavelength shift is detected by the OSA. However, as the water level increases, the amount of wavelength shift linearly increases due to the increased internal water pressure in the water tank. The wavelength multiplexed signals reflected from the SUs are then interrogated by the OSA according to the dedicated wavelength range.

Generally, the CWDM-based water level monitoring system features an environmental robustness thanks to the FBGs that have high linearity [30]. Moreover, particularly in Marques et al. [37], the temperature-induced wavelength shifts in the individual SUs are automatically compensated due to the multiplexed mPOFBGs. However, the FBGs require enough guard bands to avoid crosstalk between the SUs, which restricts the maximum number of channels within a specific wavelength band. In addition, the transmission length is limited to several meters due to the relatively high transmission loss (7 dB/m) at the 850-nm wavelength band. The same as other techniques, it requires a reference signal for mapping the wavelength shift to the water level.

## 4. DWDM-Based Water Level Monitoring System

In this section, we further explain the passive OFS network based on DWDM, which can support more than 32 SUs within a telecom C-band. Based on the operation principle, the multi-sensing characteristics are introduced in Section 4.1, and the remote-sensing capabilities are analyzed with simulations by considering the RBS effect as increasing the transmission length in Section 4.2. In Section 4.3, a dual-path architecture is explained to overcome the RBS effect, and its analysis results are presented.

### 4.1. Operation Principle and Multi-Sensing Capability

**Figure 7:**
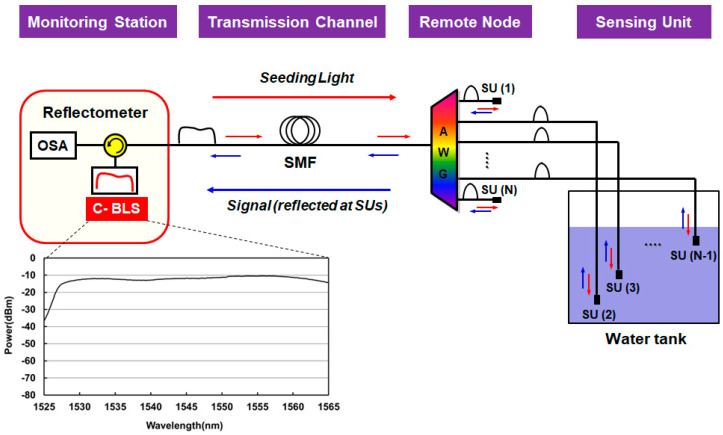
Schematic diagram of the dense wavelength division multiplexing (DWDM)-based water level monitoring system. Adapted from Lee et al. [42].

The water level monitoring system based on the DWDM technique is illustrated in Figure 7. The system is deployed in three different sections: (i) monitoring station, (ii) remote node, and (iii) water tank. In the monitoring station, the BLS in the telecom C-band (i.e., 1530–1565 nm) generates the ASE light, which is injected into the fiber-optic network via the optical circulator. After passing through the SMF, the ASE light is spectrum-sliced by the AWG in the remote node and sent to multiple channels. The number of channels and its spacing are designed according to the system requirements. Then, the spectrum-sliced ASE light of each channel is back-reflected at the SU in the water tank towards the remote node. Here, the optical reflectance at the surface of the SU is determined based on the Fresnel equations that concern the refractive indices of two junction materials ($R_{a}$: fiber-air interface and $R_{w}$: fiber-water interface), such as:

(1) $$R_{a}={\left(\frac{n_{f}-n_{a}}{n_{f}+n_{a}}\right)}^{2},\;R_{w}={\left(\frac{n_{f}-n_{w}}{n_{f}+n_{w}}\right)}^{2}$$

where $n_{f}$, $n_{a}$, and $n_{w}$ represent the refractive index of the fiber core (1.4492 at 1.55 μm), the air (1.0002739), and the water (1.3142 at 10 °C), respectively [43]. At room temperature, the actual values of $R_{a}$ and $R_{w}$ are −14.7 dB and −26.3 dB, the extinction ratio (ER) being 11.6 dB. Here, the ER means the power difference between those Fresnel reflections from the air ($R_{a})$ and the water ($R_{w})$. Thus, Equation (1) reveals that one can recognize the position of the SU (i.e., whether it is in the air or in the water) through measuring the power of the back-reflected light. The back-reflected light at multiple channels (at different wavelengths) are multiplexed by AWG and then transmitted to the monitoring station. For measuring the actual water level, the SUs are installed at different depths with even spacing (which defines a spatial resolution of this system), as seen in Figure 7.

**Figure 8:**
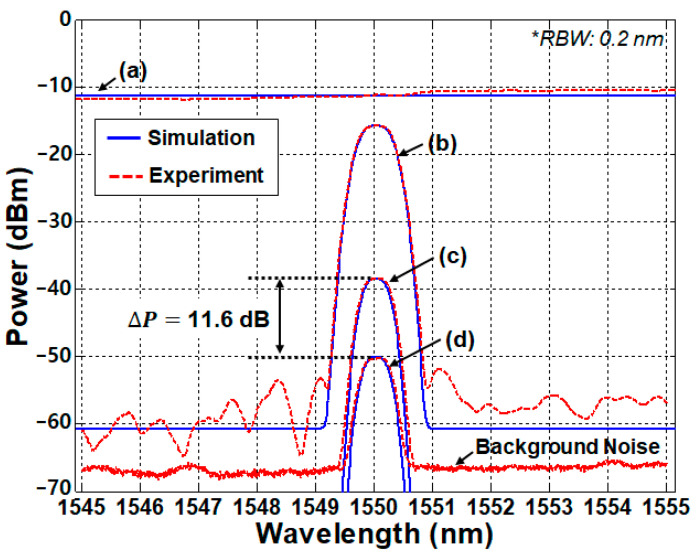
Measured/simulated optical spectrum: (**a**) the filtered amplified spontaneous emission (ASE) light, (**b**) spectrum-sliced ASE light, (**c**) back-reflected signal when the SU is in the air, and (**d**) back-reflected signal when the SU is in the water. Adapted from Lee et al. [42]. RBW: resolution bandwidth.

Figure 8 shows results of the proof-of-concept test based on the experiment, as well as the simulation, using the model proposed by Lee et al. [42,43]. The bandwidth of the filtered ASE light was 10 nm (1545–1555 nm), supporting 11 channels, with the total optical power of +6 dBm (−11.2 dBm/0.2 nm) (Figure 8a). The channel spacing and 3-dB bandwidth of the AWG were 0.8 nm (100 GHz) and 0.64 nm (80 GHz), respectively, which determines the shape of the spectrum-sliced ASE light (Figure 8b). The power difference at the center of the channel (i.e., at 1550 nm) between Figure 8a,b is attributed to the insertion loss of the AWG (~4.5 dB). Figure 8c,d shows the optical spectrum of the received back-reflected light (at the monitoring station) in two different situations: (i) the SU in the air and (ii) in the water, respectively, with the ER being measured to be ~11.6 dB, as estimated in Equation (1). The background noise in Figure 8 comes from the optical crosstalk at the circulator, as well as reflections at the fiber-optic connection points.

**Figure 9:**
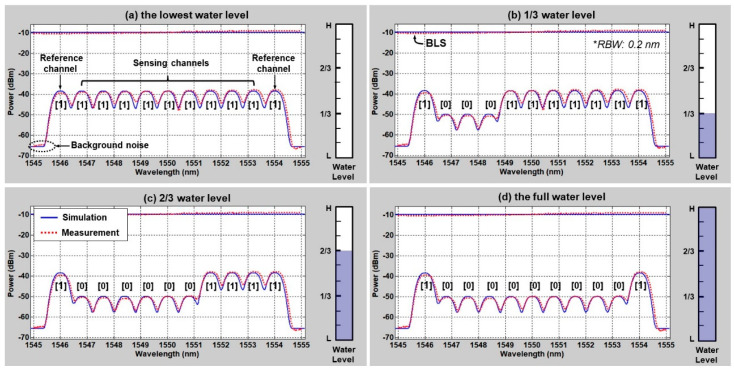
Water level measurement spectrum for four different water levels: (**a**) empty (0 height steps), (**b**) 1/3-full (3 height steps), (**c**) 2/3-full (6 height steps), and (**d**) full (9 height steps). Adapted from Lee et al. [50].

The operation principle described in Figure 8 can be extended to multiple channels to measure the water level. Figure 9 shows the four different water levels measured in back-to-back configurations. Ch#1 and #11 are references; their SUs are always in the air for a comparison to the sensing channels (Ch#2 to #10). The use of nine sensing channels allows to measure the water level in ten height steps that can be further increased by using broader bandwidths (i.e., >10 nm) of ASE light and/or narrower channel spacing (i.e., <0.8 nm). In Figure 9, “1” represents the SU in the air, while “0” represents the SU in the water.

Thus, the level of water could be represented with nine digit codes by utilizing the nine sensing channels (Ch#2 to Ch#10): e.g., empty (zero height steps: “111 111 111”) and full (nine height steps: “000 000 000”). As one can see, this system requires an interrogator, such as the OSA, though it could be replaced with simple/less-costly tools, such as an OPM, as will be discussed later.

The DWDM-based fiber-optic sensing system would suffer from a degradation of the ER due to various factors [42]. Firstly, the optical components (such as the BLS, AWG, SUs, etc.) would have the channel-dependent power/loss variations, the total value supposed to be <3.5 dB over the whole C-band. In addition, the harsh environmental conditions (such as high temperature) of the water tank may cause the index change of the water and the core of the optical fiber by $-8\times 10^{-5}/$°C and $-8.6\times 10^{-6}/$°C, respectively. To be specific, this leads to reflectance change by <0.5 dB for the range of 20–80 °C. Considering these degradation factors, the system requires at least a 5-dB power margin between the “1” level signal and the “0” level signal [42]. The specific power margin of 5 dB includes: the BLS output spectrum deviation (2.5 dB), temperature-dependent variation (0.5 dB), insertion loss variation among different channels of the AWG and SU (1 dB), and the additional power margin of 1 dB. This additional power margin is dependent on the application area and its environmental situation. Note that the “0”-level signal is supposed to be larger than the background noise.

### 4.2. Remote-Sensing Capability

**Figure 10:**
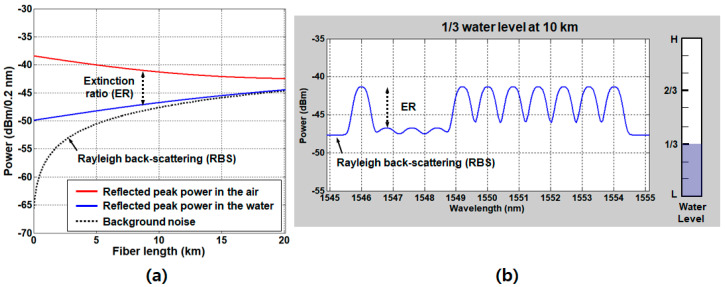
(**a**) Received optical power density as a function of the fiber length, where the RBS power approaches the signal power, and (**b**) the optical spectrum of the 1/3-full water level (3 height steps) at a 10-km fiber length. Adapted from Lee et al. [50].

In this section, we introduce the impacts of the Rayleigh back-scattering (RBS) on the performance of the DWDM-based fiber-optic sensing system. The RBS of the ASE light at the SMF is added to the signal, being a noise. First of all, the RBS coefficient ($R_{RBS}$) at the telecom C-band can be represented as [49,58]:

(2) $$R_{RBS}=\{\begin{array}{ll} -32-10\log_{10}\left(\frac{20}{D}\right), & D<20\;km\\ -32, & D\geq 20\;km \end{array}$$

where D is the SMF length in kilometers. Considering the RBS, the signal power density ($P_{1}$ and $P_{0}$ per 0.2 nm) at the center wavelength of each channel can be expressed as:

(3) $$P_{1}=PSD_{ASE}\left(10^{R_{a}/10}\times {\left(\gamma_{SMF}\times \gamma_{AWG}\times \gamma_{Cir}\right)}^{2}+10^{R_{RBS}/10}\right)$$

(4) $$P_{0}=PSD_{ASE}\left(10^{R_{w}/10}\times {\left(\gamma_{SMF}\times \gamma_{AWG}\times \gamma_{Cir}\right)}^{2}+10^{R_{RBS}/10}\right)$$

where $PSD_{ASE}$ is the power spectral density (per 0.2 nm) of the ASE light in front of the circulator. $\gamma_{Cir}$, $\gamma_{AWG}$, and $\gamma_{SMF}$ are attenuation factors (<1) of the circulator, AWG, and SMF, respectively. $\gamma_{SMF}$ is $10^{-0.2D/10}$ under an assumption that the SMF induces 0.2 dB/km attenuation. Then, the ER (i.e., $P_{1}/P_{0}$) of the signal is decreased as the fiber length increases, as seen in Figure 10a.

The power of RBS light approaches the level of the signal power as the fiber length increases. Figure 10b shows the optical spectrum of the one-third-full water level at a 10-km fiber length, simulated based on the model in Lee et al. [50]. Figure 10 reveals that the RBS noise becomes comparable to $P_{0}$ at the SMF length of 10 km, the level of ER being about 5 dB, which is the required minimum margin, as we defined in the previous section. Thus, the maximum reach of the system is limited to 10 km or less.

**Figure 11:**
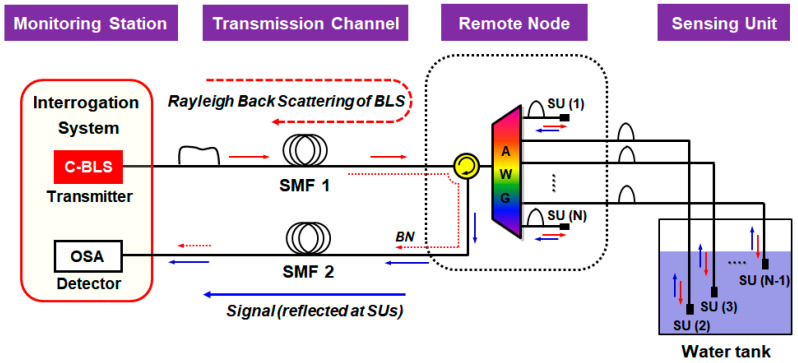
A DWDM-based water level sensing system reconfigured to mitigate the effects of Rayleigh back-scattering (RBS). Adapted from Lee et al. [50].

A simple reconfiguration of the system allows an effective mitigation of the RBS effects. The modified architecture utilizes two different optical paths, one for seeding the ASE light to the network and the other for receiving the signal from the SUs, as seen in Figure 11.

**Figure 12:**
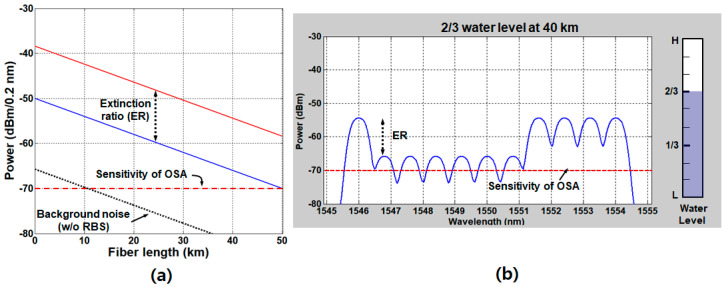
(**a**) Received optical power density as a function of the fiber length after the RBS effect is mitigated, and (**b**) the optical spectrum of the 2/3-full water level (6 height steps) at a 40-km fiber length. Adapted from Lee et al. [50].

For this, the circulator needs to be installed in the remote node. Consequently, the RBS effect is completely avoided, the ER being maintained as seen in Figure 12a.

The background noise, attributed to the crosstalk at the optical circulator (typically, −56 dB), is also decreased as the fiber length is increased due to the attenuation by the SMF2. Figure 12b shows the simulated optical spectrum in the case of the two-thirds-full water level at the SMF length of 40 km. In this configuration, the performance is mainly limited by the measuring instrument—to be more specific, the sensitivity of the OSA, which was about −70 dBm at the resolution of 0.2 nm and the sweep time of 200 ms [50]. The level of sensitivity could be enhanced at the expense of the sweep time, which may make the real-time operation unavailable. Considering that the “0” level power is supposed to be higher than the OSA sensitivity by 3 dB, the available maximum length is about 42.5 km.

### 4.3. Water Level Monitoring Based on the Optical Power Measurement

The water level monitoring based on the optical spectrum analysis requires an instrument (i.e., OSA) that is costly and needs a relatively long sweep time for increasing the number of SUs (e.g., >200 ms). This could be resolved through alternatively employing an optical power measurement method with, e.g., an OPM that operates based on the photodetectors (such as PIN-PD). The power measurement scheme is cost-effective and, ideally, offers very fast (e.g., sub-millisecond) measurement speeds (as it does not need the wavelength sweeping). Additionally, it could be exploited when the fiber-optic link between the monitoring station and the water tank is physically disconnected [43]. For example, an operator can easily access the remote node with the handheld OPM to measure the optical power and to estimate the water level for the emergency response.

**Figure 13:**
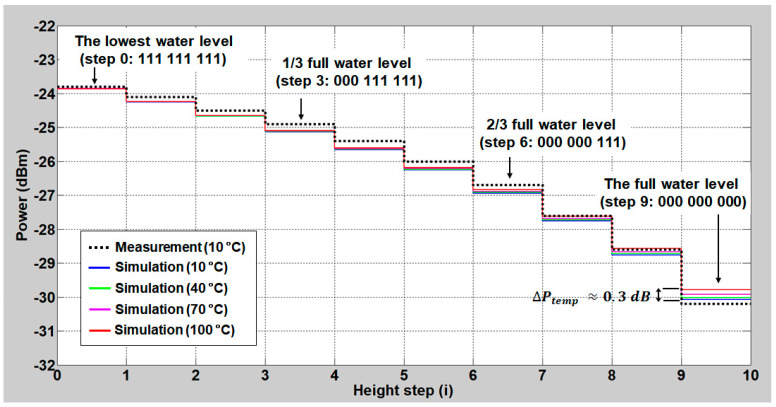
Total optical power as a function of the water level (black dashed line: measured values and solid lines: simulated results at various temperatures).

Figure 13 shows the measured/simulated received optical power (at the monitoring station) as a function of the water level in the back-to-back conditions on a decibel scale. The level of optical power is in a linear relation with the water level at a determination coefficient ($R^{2}$) of 0.998 [43]. However, there exists errors between the measured values and the theoretical curve that are less than 0.3 dB. This comes from the channel-dependent insertion loss variation of the used components and nonlinearity of the used OPM. The temperature-dependent water index variation (e.g., 1.3142 at 10 °C vs. 1.2977 at 100 °C) induces additional errors, which is negligible at the lowest water level [43]. However, this error increases as the water level rises, though it is still less than 0.5 dB.

In this power measurement scheme, the receiver reads the optical power of the whole spectrum over the DWDM channels. Thus, if there is any optical back-scattering/reflection of ASE light at the fiber-optic network, including RBS, it will be a noise, which becomes problematic in a remote-sensing situation. Thus, we analyzed the impacts of back-scattering on the performance of the power measurement scheme. The received total optical power ($P_{rx}$) can be expressed as:

(5) $$P_{rx}\left(i\right)=P_{ASE}\left\{\left(i\times 10^{\frac{R_{w}}{10}}+\left(N-i\right)\times 10^{\frac{R_{a}}{10}}\right){\left(\gamma_{SMF}\times \gamma_{AWG}\times \gamma_{Cir}\right)}^{2}+10^{\frac{R_{RBS}}{10}}+10^{\frac{R_{N}}{10}}\right\},$$

where $P_{ASE}$ is the total power of ASE light in front of the optical circulator (in microwatt), $R_{N}$ indicates the unwanted reflection components, including the crosstalk at the circulator, the two latter terms being the “noise”. The first two terms in Equation (5) indicate the “signal”, where i and N are the number of SUs submerged in the water, and total number of SUs, respectively.

Then, the signal power ratio ($\Delta P_{SPR}$) between two adjacent height steps needs to be large enough to distinguish two different water levels, which can be represented as:

(6) $$\Delta P_{SPR}\left(i\right)=\frac{P_{sig}\left(i-1\right)}{P_{sig}\left(i\right)},\;i=1,\;2,\;\cdots ,\;N$$

**Figure 14:**
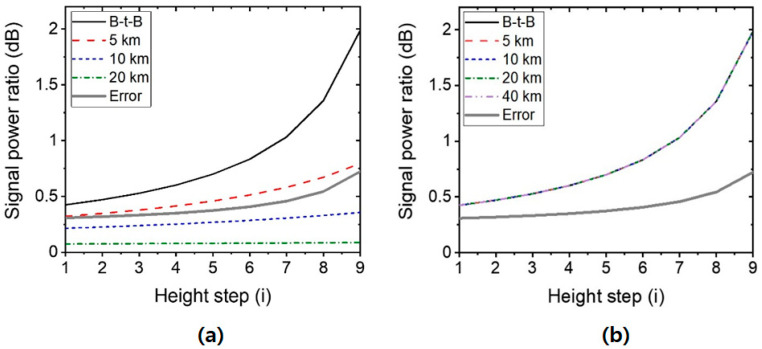
Calculated signal power ratio (SPR) as a function of the height steps for various fiber lengths: (**a**) in loop-back architecture and (**b**) dual-paths architecture, where the RBS effect is eliminated. B-t-B: Back-to-Back.

Figure 14 shows the calculated SPR ($\Delta P_{SPR}$) as a function of the water level for various fiber lengths, where the thick gray line means the error. The level of error is determined by the sum of the aforementioned errors: (i) channel-dependent losses and nonlinearity in the power (0.3 dB) and (ii) the temperature-dependent index of the water by which the received power changes (<0.5 dB). Thus, if the SPR of the system is smaller than the error, it is impossible to read the water level accurately from the received optical power. Figure 14a indicates that the distance could be only a few km, which is mainly limited by the RBS at the SMF. Therefore, the dual-paths architecture, explained in Figure 11, will be necessary for the remote sensing, as Figure 14b indicates.

With using two separate optical paths, the SPR ($\Delta P_{SPR}$) becomes independent of the fiber length, even up to 40 km. However, in this case, the performance will be limited by the sensitivity of the OPM. The calculated received optical power at 40 km was higher than −50 dBm, which is well above the OPM sensitivity (e.g., −60 dBm).

## 5. Discussion and Conclusions

As mentioned in the previous sections, the multiplexing and remote-sensing capabilities are key factors of the OFS network that we need to consider for realizing the practical water level monitoring system. In this section, some additional considerations are discussed.

The first issue is a self-protection function. The multiplexed OFS network may include tens of SUs that would be supported by a SMF of tens of kilometers. Thus, a physical damage of the SMF due to external events will result in the loss of the signal that contains the water level information, leading to the loss of proper/immediate reactions against the unwanted events. Thus, the remote optical sensor network is required to have self-protection functions for high reliability, especially in some specific applications such as a nuclear power plant. One of the simple solutions is employing 1 × 2 optical switches at the monitoring station with two SMFs, one of those being a back-up path [59].

Self-referencing is another crucial characteristic to maintain the system performance. In diverse modulation schemes using intensity, frequency, and phase, the changes of the environmental conditions can be recognized based on a comparison to the reference values provided by the optical fiber sensor network, which is called self-referencing. The self-referencing technique has been realized based on various methods, such as temporal separation, wavelength separation, spatial separation, and so on [30]. The DWDM-based sensor network described in Section 4 is an example of the wavelength separation technique, in which two wavelength channels (#1 and #N) are utilized as references (Lee et al. [42,43,50]).

In addition, the management strategy for SUs also needs be considered. For the contact-type SUs, the system performance can be degraded due to the floating particles or small particles dissolved in the water. For example, algae in a reservoir can cause a serious problem to the SUs. These contamination materials can be stuck on the surfaces of SUs over time, possibly providing false information by showing a higher water level than the actual one. Thus, appropriate plans need to be set up to manage the SUs. To solve this problem, several solutions were proposed. One is regularly replacing the contaminated SUs with a new one. For this, each SU needs to be divided into two sections with optical connectors by the segmentation method, as discussed in Lee et al. [43]. The optical connectors are then managed in the terminal box adjacent to the water tank or reservoir. Replacing the last mile section that includes the SUs on a regular basis will help to guarantee system reliability. We believe the maintenance activities such as a periodic performance test will be helpful to monitor the condition of the SUs for determination of the replacement cycle. Next, the use of a shielding case will help to prevent the fiber surfaces from the contamination [50]. The shielding case is supposed to have several small holes for the water to come in and drain out. Thus, the use of shielding cases for SUs will highly reduce the effects of mist or algae while sensing the water level properly. Lastly, the condensed water (a drop of water) attached on the end facet of the SU can be managed with the adjustment of the installation angle of each SU [50]. By installing the SUs obliquely with a certain angle (or in a horizontal direction), not in a vertical direction, the drop of water can be easily removed due to the decrease of surface tensile force of the water drop.

In conclusion, the water level-sensing system implemented with OFS networks offers various advantages that are attributed to the low loss and large bandwidth nature of the fiber-optic/photonics components. Especially, the use of multiple discrete sensors (i.e., SUs) in the OFS network (so-called quasi-distributed sensors) enables remote sensing (where its distance could be tens of kilometers) with enhanced spatial resolutions. This, however, requires optical multiplexing technologies that are being frequently utilized in telecoms for increasing the transmission capacity based on diverse physical parameters: wavelength, time, coherence, space, etc. Among those, the DWDM technique maximizes the use of an ultra-wide optical spectrum, enabling multi-channel OFS networks. In this paper, we reviewed the DWDM-based OFS network for a water level monitoring system. To be more specific, we presented the operation principle and characteristics of the DWDM OFS network that utilized the seeded ASE light with loop-back structure. Based on the optical spectrum analysis, the DWDM OFS network could measure the water level with >10 height steps at the place where it is >40 km away from the measurement field. The signal processing method (i.e., using an optical spectrum analyzer) could be further simplified so the system works with using the optical power measurement scheme, which would offer a shorter processing time as well. We also investigated that the optical power measurement scheme could also be used for the >40-km reach system. Moreover, other important system factors (such as protection, self-referencing, and the management of SUs) were discussed, manifesting itself as a highly practical solution for the remote water level monitoring system.

## Figures and Tables

**Table 1:** Examples of discrete optical fiber sensor networks based on various specialty fibers for water level monitoring.

Year/Reference	Interrogation	No. of SUs	Type of SU	Modulation Mechanism
2007/[22]	LED ^1^ (@ 660 nm) + OPM ^2^	8	U-shape bending sensor	Intensity
2007/[23]	SLS ^3^ (@ 632.8 nm) + OPM	18	Polished lateral spire sensor	Intensity
2014/[26]	SLS (@ 660 nm) + OPM	7	POF segments	Intensity
2015/[27]	LED (@ 660 nm) + OPM	26	Engraved grooves in POF	Intensity
2015/[25]	SLS (@ 633 nm) + OPM	8	Micro holes in POF	Intensity
2016/[28]	LED (@ 670 nm) + OPM	10	Grooves in POF	Intensity
2017/[20]	SLS (@ 653 nm) + OPM	30	Race-track helical POF	Intensity
2018/[29]	SLS (@ 635 nm) + OPM	20	V-grooves in POF	Intensity
2019/[21]	SLS (@ 635 nm) + OPM	20	Multi S-bend POF	Intensity
2020/[24]	SLS (@ 635 nm) + OPM	6	Screw-shaped POF	Intensity

^1^ LED: light-emitting diode, ^2^ OPM: optical power meter, ^3^ SLS: single light source (Laser), and POF: plastic optical fiber.

**Table 2:** State-of-the-art of multiplexed optical fiber sensor networks for water level monitoring.

Year/Reference	Interrogation	Multiplexing	No. of Channel	Type of SU	Modulation Mechanism
2001/[31]	OTDR	TDM	2	Right angle prism probe (R ^5^)	Intensity
2004/[32]	LD array + OPM	SDM (+FDM)	8	POF sensor head (T ^6^)	Intensity
2005/[33]	BLS + OSA ^1^	CWDM	4	FBG array (R)	Wavelength
2010/[34]	TLS ^2^ + OSA	CWDM	2	105/125 MMF ^7^ (R)	Intensity
2011/[35]	OTDR	TDM	6	Bending loss sensor (R)	Intensity
2014/[36]	OTDR	TDM	3	Special probe with NaCl solution (R)	Intensity
2015/[37]	BLS + OSA	CWDM	5	FBG array (R)	Wavelength
2015/[38]	OFDR ^3^	TDM	2	Thin core fiber (R)	Intensity
2017/[39]	OCDR ^4^	TDM	10	FC ^8^-type Fiber Connector (R)	Intensity
2018/[40]	TLS + OSA	CWDM	2	FBG-embedded diaphragm sensor (R)	Wavelength
2019/[41]	BLS + OPM	SDM	4	Cleaved end facet SMF (R)	Intensity
2019/[42]	BLS + OSA	DWDM	11	Optical patch cord (R)	Intensity
2020/[43]	BLS + OPM	DWDM	11	Optical patch cord (R)	Intensity

^1^ OSA: optical spectrum analyzer, ^2^ TLS: tunable light source, ^3^ OFDR: optical frequency domain reflectometry, ^4^ OCDR: optical coherence domain reflectometry, ^5^ R: reflective sensing type, ^6^ T: transmissive sensing type, ^7^ MMF: multimode fiber, ^8^ FC: Ferrule Connector, OTDR: optical time-domain reflectometer, LD: laser diode, BLS: broadband light source, TDM: time division multiplexing, SDM: space division multiplexing, FDM: frequency division multiplexing, DWDM: dense wavelength division multiplexing, CWDM: coarse wavelength division multiplexing, FBG: fiber Bragg grating, and SMF: single-mode fiber.
